# Effectiveness and safety of electroacupuncture for ankylosing spondylitis: A protocol for a systematic reviews and meta-analysis

**DOI:** 10.1097/MD.0000000000031706

**Published:** 2022-11-11

**Authors:** Shenghui Liu, Jiangxia Zheng, Xiuzhen Wen, Qun Fang, Xifeng Zhang

**Affiliations:** a Jiujiang First People’s Hospital Rheumatology Department, Jiujiang, Jiangxi, China.

**Keywords:** ankylosing spondylitis (AS), electroacupuncture (EA), meta-analysis

## Abstract

Ankylosing spondylitis (AS) is a type of autoimmune disease commonly characterized by joint pain and stiffness. This disease progression can exhibit common deformities and other activities with limited symptoms and significantly impacts people’s work and life. Electroacupuncture (EA) as a traditional Chinese therapy, showing several advantages (e.g., safety, economy, and less side effects), has been extensively used to treat AS. However, its curative effect is supported by limited evidence. Accordingly, the present study aims to comprehensively assess the reliability of EA in AS treatment.

**Methods and analysis:**

The study will conduct a systematic review and meta-analysis. Seven databases, including the Embase, Cochrane Library, Pubmed, SinoMed, CNKI, VIP, and Wanfang Data, will be searched using predefined search terms to identify relevant studies. The primary outcomes will be the clinical efficiency, the Bath AS Disease Activity Index, and the Visual Analog Scale. Eligible studies should report at least 1 of these primary outcomes. Eligible studies designed as randomized controlled trials will be included for meta-analysis, while other related clinical studies will be reviewed. The methodological quality of the included studies will be assessed with a previously established checklist. The Cochrane Collaboration’s bias risk tool will be used for assessing the bias of included randomized controlled trials. Stata 17.0 software is used for meta-analysis.

**Results:**

The protocol will be used to assess the efficacy and safety of EA in AS treatment.

**Conclusion:**

This review reliably evidences whether EA is a reliable method for the intervention of AS.

## 1. Introduction

Ankylosing spondylitis (AS) is a chronic, inflammatory rheumatic disease that mainly affects the axial skeleton and sacroiliac joints.^[1,2]^ The disease affects about 0.1% to 1.4% of the population, depending on the geographical region.^[3‐5]^ Besides, men are more affected than women, with a ratio of 2–3:1.^[6‐8]^ According to the report, AS patients are more prone to anxiety, depression, and other psychological problems, significantly impacting patients’ daily life.^[9‐11]^ Moreover, patients with AS not only have substantial sick leave but also experience restrictions while being at work, which led to a significant increase in the social burden.^[12]^

Currently, various drug therapy including nonsteroidal anti-inflammatory drugs (NSAIDs) and disease-modifying antirheumatic drugs (DMARDs) are exploited to prevent and treat AS.^[13–15]^ However, adverse drug reactions remain a significant challenge in these drug therapy which be popular recommend. NSAIDs have the potential to cause serious gastrointestinal, renal, and cardiovascular adverse events.^[16,17]^ DMARDs can also cause the possibility of a variety of adverse events developing in the short- and long-term.^[18–20]^ Thus, a safe and inexpensive treatment with no apparent adverse reactions should be found.

Electroacupuncture (EA) is a type of Chinese medicine therapy which is widely used as a complementary and alternative treatment for patients with AS. As a conventional non-drug therapy, it is capable of regulating Yin and Yang, activating blood circulation, and removing blood stasis. Modern studies show that EA was performed to characterize the levels of pro-inflammatory and anti-inflammatory cytokines.^[21–23]^ Although many randomized controlled trials (RCTs) have confirmed the efficacy of EA in AS treatment,^[24,25]^ it is still lack of rigorously designed systematic review and meta-analysis on EA in the treatment of AS. This review aims to assess the efficacy and safety of EA in AS treatment at home and abroad to provide evidence-based medicine for clinical practice.

## 2. Methods

### 2.1. Study registration

This protocol was developed according to the guidelines of the Cochrane Handbook for systematic reviews of interventions.^[26]^ It is registered on the International Prospective Register of Systematic Reviews. (Registration number CRD42022364925.)

### 2.2. Patient and public involvement

No patient involved.

### 2.3. Inclusion criteria for collection of studies

#### 2.3.1. *Type of study.*

It will include all RCTs of EA for AS, regardless of language or publication status. To be specific, animal trials, case studies, non-RCTs, empirical reports, and reviews will be excluded.

#### 2.3.2. *Type of participants.*

All participants satisfying the diagnostic criteria for AS as revised by the American College of Rheumatology in 1984 will be involved, regardless of race, sex, age, marital status, or educational background.

#### 2.3.3. *Type of interventions.*

Intervention measures should be EA alone or combined with other methods to treat AS. If combined with other methods, only the control group with the same intervention measures as the experimental group will be included.

#### 2.3.4. *Type of comparator (S)/control.*

None of the restrictions on the treatment options for the control group, including no therapy, placebo, or any other control considered for comparison.

#### 2.3.5. *Types of outcome measurements.*

1. Main outcomes.

Clinical efficiency, Bath AS Disease Activity Index, and visual analog scale were the primary outcomes.

1. Secondary outcomes.

①Finger-to-floor distance;

②occiput to wall distance;

③Erythrocyte sedimentation rate, C-reactive protein;

④Adverse reactions.

### 2.4. *Search strategies for recognizing studies*

1. The primary source of data.

RCTs of EA for AS will be searched till October 2022 from the Chinese Biomedical Literature Database, Chongqing VIP Database for Chinese Technical Periodicals, China National Knowledge Infrastructure, Wanfang, Web of Science, Cochrane Library, PubMed, and EMBASE. The retrieval strategies adopted by PubMed are elucidated in Table 1.

**Table 1:** Search strategy (PubMed).

Number	Search terms
#1	MeSH: “Ankylosing Spondylitis”
#2	Ti/Ab: “Ankylosing Spondylitis” OR “Spondyloarthritis” OR “Spondyloarthropathies” OR “Seronegative Spondyloarthropathies”
#3	#1 OR #2
#4	MeSH: “Electroacupuncture”
#5	Ti/Ab: “Electroacupuncture” OR “Electro acupuncture” OR “Ele-acupuncture” OR “Electro-stimulation” OR “Acupuncture” OR “Acupoint” OR “Meridians”
#6	#4 OR #5
#7	MeSH: “randomized controlled trial” OR “randomized controlled trial as Topic” OR “controlled clinical trial”
#8	Ti/Ab: “randomized controlled trial” OR “random allocation” OR “allocation” OR “haphazard” OR “RCT randomized controlled” OR “randomized” OR “controlled” OR “clinical trial”
#9	#7 OR #8
#10	#3 AND #6 AND #9

Ab = abstract, Ti = title.

1. Search of other resources.

Some unfinished or unpublished experimental data were retrieved from the Chinese Clinical Trial Registry and The Clinicaltrials.gov.

### 2.5. Data acquisition and analysis

**Figure 1.**
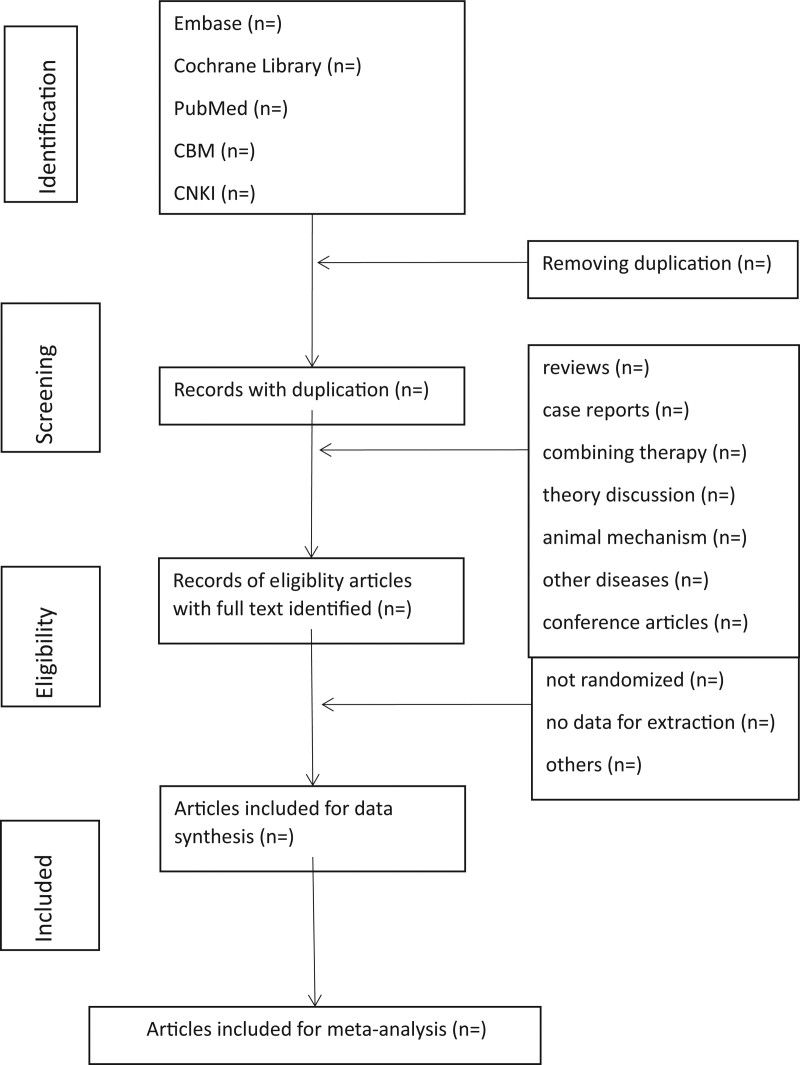
Flowchart of literature selection.

First, all the literature was imported into the EndNote X9 (Thomson Research Soft, Stamford, CT), and all duplicate literature will be deleted. Second, LSH and ZJX will be adopted to review the titles and abstracts, and the irrelevant literature will be removed. Third, the full text will be read to determine if the project will be included here. Lastly, 2 researchers (WXZ and FQ) will conduct the crosscheck. If there were disagreements, the third researcher (ZXF) would participate in the discussion and solve it. Figure 1 illustrates a flow chart of literature screening.

### 2.6. Data acquisition and management

Two researchers (WXZ and FQ) will each extract the qualified data into a pre-made table, and a third will step in to resolve any potential differences. The extracted data consisted of journal, author information, title, publication date, participant characteristics, sample size, interventions, study methods, primary and secondary outcome measures, as well as any adverse events.

### 2.7. Assessment of risk of bias in included studies

ZXF and FQ will employ the Cochrane Bias risk Assessment tool to determine the quality of the trials, respectively.^[27]^ The extracted details were as follows: the random sequence generation, the blindness of result evaluation, the blindness of participants and personnel, the concealment of allocation, the reporting of selective results, the incomplete result data, and so on. These fell to 3 levels, that is, fuzzy, low, and high. In case of ambiguity, the author of the relevant project would be contacted. If there were any disputes, an informed decision would be made with the assistance of the third investigator (WXZ).

### 2.8. Dealing with missing data

In the event of ambiguous data, we will contact the corresponding author by phone or email. We will exclude missing information from the analysis if missing information is unavailable.

### 2.9. Data synthesis and analysis

This study will use Stata 17.0 (Beijing Tianyan Rongzhi Software Co., LTD, Beijing, China). It provides all the data analysis, data management and graphics you need. The measurement data will use the mean difference (MD) as the effect indicator, and the count data will use the odds ratio (OR) as the effect index. Each effect indicator will be given as a point estimate with 95% confidence interval. The heterogeneity and size of each study result will be judged using statistical methods. For studies with no statistical heterogeneity, the analysis will be performed using a fixed-effect model, whereas a randomized effects model will be applied if for studies with significant statistical heterogeneity.

### 2.10. Subgroup analysis

If there were significant heterogeneity between the trials involved, the course and sample size, type, time, and frequency of EA would be considered for subgroup analysis.

### 2.11. Sensitivity analysis

Sensitivity analysis was conducted to exclude low-quality literature to ensure the stability and accuracy of the conclusions drawn from this meta-analysis.

### 2.12. Assessment of reporting biases

If the number of RCTs exceeded 10, funnel plot analysis would be required to test for publication bias. In addition, if there was an asymmetric funnel graph, the Egger check would be conducted to study the causes of publication bias.

### 2.13. Ethics and dissemination

This study will not involve primary data collection, and formal ethics approval will, therefore, not be required. The results from this study will be disseminated through conferences and in peer-reviewed journals.

## 3. Discussion

AS refers to an autoimmune disease that mainly affects the axial skeleton and sacroiliac joints. It can affect patients’ life and work, bringing economic and social burdens. Drug therapies are effective in treating AS, particularly NSAIDs and DMARDs, whereas there are concerns about the side effects of drug therapies. In contrast, EA, as an effective technique of traditional Chinese medicine, has been accepted for AS treatment in China. However, as far as the current study is concerned, the efficacy and safety of EA in AS treatment are not supported by data. Thus, this study is expected to evidence the clinical use of EA in AS treatment.

Some possible limitations of this study should be claimed here, including poor quality of the original study, false positive or negative results, different disease duration, different intervention doses, different intervention frequencies, language limitations, etc. These limitations may lead to certain biases and affect the evaluation results.

## Author contributions

All authors have read and approved the publication of the protocol.

**Conceptualization:** Shenghui Liu, Jiangxia Zheng.

**Data curation:** Shenghui Liu, Jiangxia Zheng, Xiuzhen Wen, Qun Fang, Xifeng Zhang.

**Formal analysis:** Jiangxia Zheng, Shenghui Liu.

**Investigation:** Shenghui Liu, Xiuzhen Wen, Jiangxia Zheng.

**Methodology:** Shenghui Liu, Xiuzhen Wen, Qun Fang, Xifeng Zhang.

**Software:** Jiangxia Zheng, Xifeng Zhang.

**Supervision:** Shenghui Liu, Jiangxia Zheng.

**Writing – original draft:** Shenghui Liu, Qun Fang, Xifeng Zhang.

**Writing** – **review and editing:** Shenghui Liu, Jiangxia Zheng, Xiuzhen Wen, Qun Fang, Xifeng Zhang.
